# Comment on “KCTD9 expression predicts immunotherapy response and enhances Anti-PD-1 efficacy in colon adenocarcinoma”

**DOI:** 10.1097/JS9.0000000000003073

**Published:** 2025-07-14

**Authors:** Zhiping Huang, Kehang Dai

**Affiliations:** Gastrointestinal Department, Chongqing University Three Gorges Hospital, Chongqing, Wanzhou, China

*Dear Editor,*

We read with great interest the recent article by Wu *et al*, titled *“KCTD9 Expression Predicts Immunotherapy Response and Enhances Anti-PD-1 Efficacy in Colon Adenocarcinoma*^[1]^*”*. The study demonstrates that KCTD9 expression is positively associated with CD8^+^ T-cell infiltration and may enhance the efficacy of anti-PD-1 therapy in colon cancer. We commend the authors for their comprehensive multi-omics approach and well-designed animal experiments. However, we would like to raise a methodological concern regarding the **choice of the MC38 murine model**, which may influence the interpretation and generalizability of the findings. We state that the manuscript was developed following the TITAN Guidelines 2025^[2]^ for the declaration and use of artificial intelligence in scholarly publishing. No AI tools were employed in the conception, design, analysis, interpretation, or writing of this work.

MC38 is a **murine colon adenocarcinoma cell line with microsatellite instability-high (MSI-H) phenotype**, characterized by high tumor mutational burden and robust immunogenicity. Prior studies have shown that MC38 tumors are **intrinsically responsive to immune checkpoint blockade**, particularly anti-PD-1 therapy, even without combination interventions^[3,4]^. Therefore, the use of this model, while valid, may **exaggerate the immunotherapeutic enhancement** attributed to KCTD9 overexpression in this context.

Importantly, **the majority of colorectal cancer (CRC) patients are microsatellite stable (MSS)** and less responsive to PD-1 blockade^[3]^. Thus, validation in MSS-type CRC models, such as CT26 murine or HT29 human, would be critical to confirm whether the synergistic effects observed with KCTD9 apply to broader patient populations.

We acknowledge that the authors mentioned the use of only one cell line as a limitation. However, they did not explicitly discuss the **inherent immunobiological bias of the MC38 model**, nor its potential to confound interpretation of KCTD9’s effect size. We believe that highlighting this point is essential, as it directly relates to the translational relevance of the findings.

We appreciate the authors’ contribution to this promising area of immunotherapy research and hope our comments may help in refining future studies toward broader clinical applicability.

## Data Availability

The letter does not have any data.

## Research registration unique identifying number (UIN)

Not registered for the type of the study.
